# 14-De­oxyxyloccensin K from *Chisocheton ceramicus* (Meliaceae)

**DOI:** 10.1107/S160053681002564X

**Published:** 2010-07-07

**Authors:** Ibrahim A. Najmuldeen, Abdul Hamid Abdul Hadi, Khalijah Awang, Khalit Mohamad, Seik Weng Ng

**Affiliations:** a Department of Chemistry, University of Malaya, 50603 Kuala Lumpur, Malaysia; b Department of Pharmacy, Faculty of Medicine, University of Malaya, 50603 Kuala Lumpur, Malaysia

## Abstract

The title limonoid 14-de­oxyxyloccensin K, C_27_H_34_O_7_, isolated from *Chisocheton ceramicus* (Meliaceae), features an oxygen linkage between carbon-3 and carbon-8 along with a tetra­hydro­furyl sub-unit. The six-membered rings adopt chair configurations and the tetra­hydro­furyl sub-unit has an envelope shape.

## Related literature

For the synthesis of 14-de­oxyxyloccensin K from xyloccensin K, see: Kim *et al.* (2004 ▶). For the crystal structure of xyloccensin K, see: Kokpol *et al.* (1996 ▶). For a description of other xyloccensin limonoids, see: Wu *et al.* (2003 ▶).
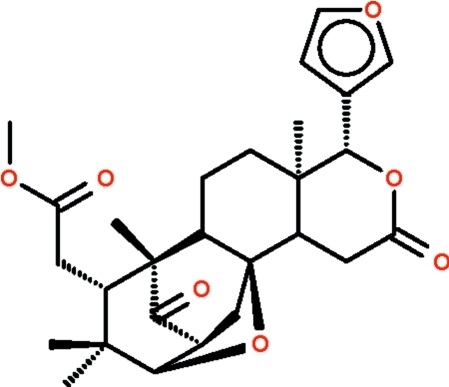


## Experimental

### 

#### Crystal data

C_27_H_34_O_7_
*M* *_r_* = 470.54
Orthorhombic, 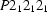

*a* = 8.7174 (6) Å
*b* = 11.7401 (8) Å
*c* = 23.1106 (16) Å
*V* = 2365.2 (3) Å^3^
*Z* = 4
Mo *K*α radiation
μ = 0.10 mm^−1^
*T* = 100 K
0.25 × 0.15 × 0.05 mm

#### Data collection

Bruker SMART APEX diffractometer
22814 measured reflections
3083 independent reflections
2674 reflections with *I* > 2σ(*I*)
*R*_int_ = 0.053

#### Refinement

*R*[*F* ^2^ > 2σ(*F* ^2^)] = 0.038
*wR*(*F* ^2^) = 0.098
*S* = 1.03
3083 reflections
307 parameters
H-atom parameters constrained
Δρ_max_ = 0.29 e Å^−3^
Δρ_min_ = −0.20 e Å^−3^

### 

Data collection: *APEX2* (Bruker, 2009 ▶); cell refinement: *SAINT* (Bruker, 2009 ▶); data reduction: *SAINT*; program(s) used to solve structure: *SHELXS97* (Sheldrick, 2008 ▶); program(s) used to refine structure: *SHELXL97* (Sheldrick, 2008 ▶); molecular graphics: *X-SEED* (Barbour, 2001 ▶); software used to prepare material for publication: *publCIF* (Westrip, 2010 ▶).

## Supplementary Material

Crystal structure: contains datablocks global, I. DOI: 10.1107/S160053681002564X/bt5284sup1.cif

Structure factors: contains datablocks I. DOI: 10.1107/S160053681002564X/bt5284Isup2.hkl

Additional supplementary materials:  crystallographic information; 3D view; checkCIF report

## Figures and Tables

**Fig. 1.**
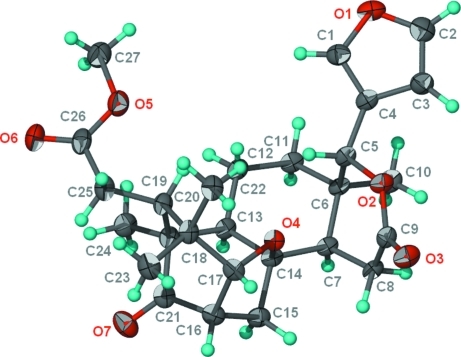
Anisotropic displacement ellipsoid plot (Barbour, 2001) of C27H34O7 at the 70% probability level; hydrogen atoms are drawn as spheres of arbitrary radius.

## supplementary crystallographic information

### Comment

The limonoid, xyloccensin K, which was isolated from the seeds of *Xylocarpus granatum*, has a structure similar to those of other limonoids but it features an ether linkage connecting the C3 and C8 atoms along with a tetrahydrofuryl sub-unit (Kokpol *et al.*, 1996). As the compound is also a tertiary alcohol, the compound can also be deoxygenated by a free-radical process (Kim *et al.*, 2004). The synthetic compound was fully characterized by spectroscopic methods. This deoxygenated compound (Scheme I, Fig. 1) was isolated from the bark of *Chisocheton ceramicus* in the present study; the spectroscopic assignments are confirmed by the crystal structure determination.

### Experimental

The bark of *C. ceramicine* (900 g), collected in Kedah, Peninsular Malaysia, was dried, ground, and extracted successively with methanol. The extract (200 g) was partitioned with 10% aqueous methanol and ethyl acetate. The ethyl acetate-soluble fraction (10 g) was subjected to silica gel column-chromatography (hexane/ethyl acetate 1:0 to 0:1). The fraction eluted with hexane/ethyl acetate (2:8) was purified on a silica gel column (ethyl acetate:acetone:hexane 65:10:25). Further HPLC purification followed by recrystallization from aqueous methanol yielded colorless crystals.

### Refinement

H-atoms were placed in calculated positions (C—H 0.95 to 1.00 Å) and were included in the refinement in the riding model approximation, with *U*(H) set to 1.2 to 1.5*U*(C).

The absolute configuration was set to that of xyloccensin K, which was isolated from the seeds of *Xylocarpus granatum* (Kokpol *et al.*, 1996).

2348 Friedel pairs were merged.

### Crystal data

**Table:**

C_27_H_34_O_7_	*F*(000) = 1008
*M**_r_* = 470.54	*D*_x_ = 1.321 Mg m^−^^3^
Orthorhombic, *P*2_1_2_1_2_1_	Mo *K*α radiation, λ = 0.71073 Å
Hall symbol: P 2ac 2ab	Cell parameters from 5382 reflections
*a* = 8.7174 (6) Å	θ = 2.5–27.9°
*b* = 11.7401 (8) Å	µ = 0.10 mm^−^^1^
*c* = 23.1106 (16) Å	*T* = 100 K
*V* = 2365.2 (3) Å^3^	Triangular plate, colorless
*Z* = 4	0.25 × 0.15 × 0.05 mm

### Data collection

**Table:**

Bruker SMART APEX diffractometer	2674 reflections with *I* > 2σ(*I*)
Radiation source: fine-focus sealed tube	*R*_int_ = 0.053
graphite	θ_max_ = 27.5°, θ_min_ = 1.8°
ω scans	*h* = −11→10
22814 measured reflections	*k* = −15→15
3083 independent reflections	*l* = −29→29

### Refinement

**Table:**

Refinement on *F*^2^	Primary atom site location: structure-invariant direct methods
Least-squares matrix: full	Secondary atom site location: difference Fourier map
*R*[*F*^2^ > 2σ(*F*^2^)] = 0.038	Hydrogen site location: inferred from neighbouring sites
*wR*(*F*^2^) = 0.098	H-atom parameters constrained
*S* = 1.03	*w* = 1/[σ^2^(*F*_o_^2^) + (0.0502*P*)^2^ + 0.7549*P*] where *P* = (*F*_o_^2^ + 2*F*_c_^2^)/3
3083 reflections	(Δ/σ)_max_ = 0.001
307 parameters	Δρ_max_ = 0.29 e Å^−^^3^
0 restraints	Δρ_min_ = −0.20 e Å^−^^3^

### Fractional atomic coordinates and isotropic or equivalent isotropic displacement parameters (Å^2^)

**Table:**

	*x*	*y*	*z*	*U*_iso_*/*U*_eq_	
O1	0.7791 (2)	0.99435 (15)	0.16955 (8)	0.0278 (4)	
O2	0.34822 (19)	0.79390 (12)	0.19484 (7)	0.0208 (4)	
O3	0.1819 (2)	0.65454 (13)	0.19079 (8)	0.0267 (4)	
O4	0.20193 (19)	0.82792 (12)	0.31200 (6)	0.0177 (3)	
O5	0.5624 (2)	1.06497 (15)	0.44621 (7)	0.0281 (4)	
O6	0.4725 (2)	1.10625 (17)	0.53467 (8)	0.0333 (5)	
O7	−0.0536 (2)	0.96552 (15)	0.46084 (8)	0.0306 (4)	
C1	0.6616 (3)	0.9897 (2)	0.20877 (11)	0.0242 (5)	
H1	0.6643	1.0232	0.2462	0.029*	
C2	0.7301 (3)	0.9356 (2)	0.12191 (12)	0.0280 (6)	
H2	0.7888	0.9241	0.0878	0.034*	
C3	0.5866 (3)	0.8964 (2)	0.13033 (11)	0.0250 (5)	
H3	0.5268	0.8537	0.1037	0.030*	
C4	0.5416 (3)	0.93121 (19)	0.18712 (10)	0.0199 (5)	
C5	0.3928 (3)	0.90631 (18)	0.21706 (10)	0.0181 (5)	
H5	0.4153	0.8975	0.2592	0.022*	
C6	0.2663 (3)	0.99724 (19)	0.21106 (10)	0.0174 (5)	
C7	0.1137 (3)	0.94872 (18)	0.23416 (10)	0.0180 (5)	
H7	0.0320	1.0060	0.2253	0.022*	
C8	0.0746 (3)	0.84036 (19)	0.20019 (10)	0.0199 (5)	
H8A	−0.0151	0.8033	0.2185	0.024*	
H8B	0.0448	0.8621	0.1604	0.024*	
C9	0.2029 (3)	0.75607 (19)	0.19700 (10)	0.0201 (5)	
C10	0.2485 (3)	1.0343 (2)	0.14753 (11)	0.0237 (5)	
H10A	0.3462	1.0648	0.1334	0.036*	
H10B	0.1692	1.0932	0.1447	0.036*	
H10C	0.2187	0.9685	0.1240	0.036*	
C11	0.3102 (3)	1.10229 (18)	0.24743 (10)	0.0196 (5)	
H11A	0.4114	1.1305	0.2343	0.024*	
H11B	0.2341	1.1633	0.2403	0.024*	
C12	0.3179 (3)	1.07930 (19)	0.31233 (10)	0.0204 (5)	
H12A	0.3410	1.1513	0.3329	0.024*	
H12B	0.4023	1.0251	0.3203	0.024*	
C13	0.1678 (3)	1.03012 (18)	0.33536 (10)	0.0187 (5)	
H13	0.0895	1.0914	0.3298	0.022*	
C14	0.1096 (3)	0.92757 (18)	0.29983 (10)	0.0182 (5)	
C15	−0.0487 (3)	0.8923 (2)	0.32228 (10)	0.0210 (5)	
H15A	−0.0979	0.8353	0.2967	0.025*	
H15B	−0.1176	0.9586	0.3271	0.025*	
C16	−0.0026 (3)	0.8406 (2)	0.38075 (10)	0.0215 (5)	
H16	−0.0819	0.7875	0.3966	0.026*	
C17	0.1497 (3)	0.77978 (19)	0.36585 (10)	0.0196 (5)	
H17	0.1292	0.6967	0.3602	0.024*	
C18	0.2718 (3)	0.79431 (19)	0.41271 (10)	0.0204 (5)	
C19	0.3077 (3)	0.92499 (19)	0.42077 (10)	0.0200 (5)	
H19	0.3949	0.9428	0.3942	0.024*	
C20	0.1715 (3)	1.00645 (19)	0.40270 (10)	0.0202 (5)	
C21	0.0274 (3)	0.9414 (2)	0.41994 (10)	0.0221 (5)	
C22	0.4198 (3)	0.7342 (2)	0.39397 (10)	0.0238 (5)	
H22A	0.3997	0.6527	0.3888	0.036*	
H22B	0.4985	0.7446	0.4238	0.036*	
H22C	0.4559	0.7669	0.3574	0.036*	
C23	0.2093 (3)	0.7351 (2)	0.46731 (10)	0.0269 (6)	
H23A	0.1894	0.6547	0.4588	0.040*	
H23B	0.1138	0.7721	0.4794	0.040*	
H23C	0.2851	0.7408	0.4985	0.040*	
C24	0.1745 (3)	1.1215 (2)	0.43403 (11)	0.0249 (5)	
H24A	0.0877	1.1680	0.4211	0.037*	
H24B	0.2707	1.1610	0.4252	0.037*	
H24C	0.1671	1.1090	0.4759	0.037*	
C25	0.3663 (3)	0.9470 (2)	0.48241 (10)	0.0245 (5)	
H25A	0.2762	0.9555	0.5081	0.029*	
H25B	0.4230	0.8785	0.4954	0.029*	
C26	0.4692 (3)	1.0497 (2)	0.49100 (10)	0.0254 (5)	
C27	0.6748 (3)	1.1553 (2)	0.45213 (12)	0.0317 (6)	
H27A	0.7375	1.1592	0.4170	0.047*	
H27B	0.7409	1.1395	0.4855	0.047*	
H27C	0.6220	1.2281	0.4579	0.047*	

### Atomic displacement parameters (Å^2^)

**Table:**

	*U*^11^	*U*^22^	*U*^33^	*U*^12^	*U*^13^	*U*^23^
O1	0.0202 (9)	0.0236 (9)	0.0395 (10)	−0.0015 (8)	−0.0016 (8)	0.0028 (8)
O2	0.0216 (9)	0.0111 (7)	0.0297 (9)	−0.0028 (7)	−0.0001 (7)	−0.0013 (6)
O3	0.0285 (10)	0.0143 (8)	0.0372 (9)	−0.0041 (8)	−0.0022 (8)	−0.0015 (7)
O4	0.0210 (9)	0.0118 (7)	0.0202 (7)	0.0020 (6)	−0.0004 (7)	0.0014 (6)
O5	0.0338 (11)	0.0255 (9)	0.0250 (9)	−0.0024 (9)	−0.0002 (8)	−0.0053 (7)
O6	0.0369 (12)	0.0394 (11)	0.0235 (9)	−0.0049 (10)	−0.0029 (9)	−0.0100 (8)
O7	0.0355 (11)	0.0244 (9)	0.0319 (10)	0.0036 (8)	0.0113 (9)	−0.0022 (7)
C1	0.0217 (12)	0.0238 (12)	0.0272 (12)	−0.0006 (10)	−0.0013 (10)	0.0037 (10)
C2	0.0304 (15)	0.0207 (12)	0.0328 (13)	0.0014 (11)	0.0034 (11)	−0.0013 (10)
C3	0.0263 (14)	0.0197 (11)	0.0290 (13)	−0.0043 (10)	0.0019 (11)	−0.0025 (10)
C4	0.0211 (12)	0.0140 (10)	0.0248 (11)	−0.0005 (9)	−0.0033 (10)	0.0020 (9)
C5	0.0214 (12)	0.0101 (10)	0.0228 (11)	−0.0021 (9)	−0.0019 (9)	−0.0004 (8)
C6	0.0165 (11)	0.0131 (10)	0.0225 (11)	−0.0015 (9)	−0.0041 (9)	0.0019 (9)
C7	0.0180 (12)	0.0123 (10)	0.0237 (11)	−0.0006 (9)	−0.0036 (9)	0.0014 (8)
C8	0.0194 (12)	0.0173 (11)	0.0231 (12)	−0.0026 (9)	−0.0046 (9)	−0.0013 (9)
C9	0.0241 (13)	0.0169 (10)	0.0191 (11)	−0.0025 (9)	−0.0024 (10)	0.0014 (8)
C10	0.0268 (13)	0.0189 (11)	0.0255 (12)	−0.0012 (10)	−0.0043 (10)	0.0044 (9)
C11	0.0219 (13)	0.0097 (9)	0.0272 (11)	−0.0011 (9)	−0.0026 (10)	0.0025 (8)
C12	0.0237 (12)	0.0130 (10)	0.0246 (11)	−0.0029 (9)	−0.0017 (10)	−0.0029 (9)
C13	0.0217 (12)	0.0103 (9)	0.0241 (11)	0.0002 (9)	−0.0016 (9)	−0.0024 (8)
C14	0.0178 (11)	0.0114 (9)	0.0253 (12)	0.0010 (9)	−0.0014 (9)	−0.0005 (8)
C15	0.0207 (12)	0.0136 (10)	0.0286 (12)	−0.0022 (9)	0.0003 (10)	−0.0010 (9)
C16	0.0227 (13)	0.0149 (10)	0.0269 (12)	0.0005 (10)	0.0026 (10)	0.0002 (9)
C17	0.0237 (13)	0.0117 (10)	0.0234 (11)	0.0009 (9)	0.0030 (10)	0.0017 (8)
C18	0.0269 (13)	0.0148 (10)	0.0196 (11)	0.0034 (10)	−0.0006 (9)	−0.0005 (8)
C19	0.0237 (13)	0.0165 (10)	0.0198 (11)	0.0012 (10)	−0.0018 (10)	−0.0023 (9)
C20	0.0231 (13)	0.0159 (10)	0.0218 (11)	0.0018 (10)	−0.0005 (10)	−0.0026 (9)
C21	0.0278 (13)	0.0149 (11)	0.0236 (12)	0.0044 (10)	0.0000 (10)	0.0003 (9)
C22	0.0282 (14)	0.0195 (11)	0.0237 (12)	0.0063 (10)	−0.0016 (10)	−0.0007 (9)
C23	0.0343 (15)	0.0215 (12)	0.0248 (12)	0.0038 (11)	0.0024 (11)	0.0024 (10)
C24	0.0270 (14)	0.0171 (11)	0.0307 (13)	0.0028 (10)	−0.0005 (11)	−0.0055 (9)
C25	0.0306 (14)	0.0226 (12)	0.0203 (11)	0.0024 (11)	−0.0016 (10)	−0.0013 (9)
C26	0.0287 (14)	0.0279 (12)	0.0197 (11)	0.0055 (11)	−0.0058 (10)	−0.0020 (10)
C27	0.0345 (16)	0.0297 (13)	0.0308 (13)	−0.0056 (13)	−0.0013 (12)	−0.0045 (11)

### Geometric parameters (Å, °)

**Table:**

O1—C2	1.368 (3)	C12—H12B	0.9900
O1—C1	1.369 (3)	C13—C14	1.543 (3)
O2—C9	1.343 (3)	C13—C20	1.581 (3)
O2—C5	1.468 (3)	C13—H13	1.0000
O3—C9	1.215 (3)	C14—C15	1.531 (3)
O4—C17	1.441 (3)	C15—C16	1.535 (3)
O4—C14	1.448 (3)	C15—H15A	0.9900
O5—C26	1.328 (3)	C15—H15B	0.9900
O5—C27	1.450 (3)	C16—C21	1.514 (3)
O6—C26	1.209 (3)	C16—C17	1.546 (3)
O7—C21	1.214 (3)	C16—H16	1.0000
C1—C4	1.348 (3)	C17—C18	1.528 (3)
C1—H1	0.9500	C17—H17	1.0000
C2—C3	1.347 (4)	C18—C22	1.533 (3)
C2—H2	0.9500	C18—C23	1.540 (3)
C3—C4	1.430 (3)	C18—C19	1.577 (3)
C3—H3	0.9500	C19—C25	1.535 (3)
C4—C5	1.499 (3)	C19—C20	1.580 (3)
C5—C6	1.541 (3)	C19—H19	1.0000
C5—H5	1.0000	C20—C21	1.523 (4)
C6—C10	1.539 (3)	C20—C24	1.533 (3)
C6—C11	1.541 (3)	C22—H22A	0.9800
C6—C7	1.543 (3)	C22—H22B	0.9800
C7—C8	1.533 (3)	C22—H22C	0.9800
C7—C14	1.538 (3)	C23—H23A	0.9800
C7—H7	1.0000	C23—H23B	0.9800
C8—C9	1.495 (3)	C23—H23C	0.9800
C8—H8A	0.9900	C24—H24A	0.9800
C8—H8B	0.9900	C24—H24B	0.9800
C10—H10A	0.9800	C24—H24C	0.9800
C10—H10B	0.9800	C25—C26	1.516 (4)
C10—H10C	0.9800	C25—H25A	0.9900
C11—C12	1.525 (3)	C25—H25B	0.9900
C11—H11A	0.9900	C27—H27A	0.9800
C11—H11B	0.9900	C27—H27B	0.9800
C12—C13	1.526 (3)	C27—H27C	0.9800
C12—H12A	0.9900		
			
C2—O1—C1	106.2 (2)	C14—C15—C16	99.77 (19)
C9—O2—C5	122.25 (18)	C14—C15—H15A	111.8
C17—O4—C14	108.00 (17)	C16—C15—H15A	111.8
C26—O5—C27	116.02 (19)	C14—C15—H15B	111.8
C4—C1—O1	110.8 (2)	C16—C15—H15B	111.8
C4—C1—H1	124.6	H15A—C15—H15B	109.5
O1—C1—H1	124.6	C21—C16—C15	105.23 (19)
C3—C2—O1	110.3 (2)	C21—C16—C17	110.2 (2)
C3—C2—H2	124.9	C15—C16—C17	102.18 (18)
O1—C2—H2	124.9	C21—C16—H16	112.8
C2—C3—C4	106.8 (2)	C15—C16—H16	112.8
C2—C3—H3	126.6	C17—C16—H16	112.8
C4—C3—H3	126.6	O4—C17—C18	110.38 (19)
C1—C4—C3	105.9 (2)	O4—C17—C16	106.42 (18)
C1—C4—C5	126.9 (2)	C18—C17—C16	112.89 (19)
C3—C4—C5	127.3 (2)	O4—C17—H17	109.0
O2—C5—C4	104.05 (18)	C18—C17—H17	109.0
O2—C5—C6	113.69 (18)	C16—C17—H17	109.0
C4—C5—C6	116.28 (18)	C17—C18—C22	109.56 (19)
O2—C5—H5	107.5	C17—C18—C23	106.5 (2)
C4—C5—H5	107.5	C22—C18—C23	108.74 (19)
C6—C5—H5	107.5	C17—C18—C19	109.30 (18)
C10—C6—C11	108.62 (18)	C22—C18—C19	108.3 (2)
C10—C6—C5	110.7 (2)	C23—C18—C19	114.36 (19)
C11—C6—C5	109.13 (18)	C25—C19—C18	109.83 (19)
C10—C6—C7	110.34 (19)	C25—C19—C20	113.17 (19)
C11—C6—C7	108.73 (19)	C18—C19—C20	114.12 (19)
C5—C6—C7	109.28 (18)	C25—C19—H19	106.4
C8—C7—C14	111.49 (18)	C18—C19—H19	106.4
C8—C7—C6	108.70 (19)	C20—C19—H19	106.4
C14—C7—C6	114.90 (19)	C21—C20—C24	109.4 (2)
C8—C7—H7	107.1	C21—C20—C19	104.29 (18)
C14—C7—H7	107.1	C24—C20—C19	113.3 (2)
C6—C7—H7	107.1	C21—C20—C13	109.2 (2)
C9—C8—C7	114.10 (19)	C24—C20—C13	108.09 (18)
C9—C8—H8A	108.7	C19—C20—C13	112.45 (19)
C7—C8—H8A	108.7	O7—C21—C16	123.2 (2)
C9—C8—H8B	108.7	O7—C21—C20	124.5 (2)
C7—C8—H8B	108.7	C16—C21—C20	112.2 (2)
H8A—C8—H8B	107.6	C18—C22—H22A	109.5
O3—C9—O2	117.6 (2)	C18—C22—H22B	109.5
O3—C9—C8	122.8 (2)	H22A—C22—H22B	109.5
O2—C9—C8	119.23 (19)	C18—C22—H22C	109.5
C6—C10—H10A	109.5	H22A—C22—H22C	109.5
C6—C10—H10B	109.5	H22B—C22—H22C	109.5
H10A—C10—H10B	109.5	C18—C23—H23A	109.5
C6—C10—H10C	109.5	C18—C23—H23B	109.5
H10A—C10—H10C	109.5	H23A—C23—H23B	109.5
H10B—C10—H10C	109.5	C18—C23—H23C	109.5
C12—C11—C6	113.94 (18)	H23A—C23—H23C	109.5
C12—C11—H11A	108.8	H23B—C23—H23C	109.5
C6—C11—H11A	108.8	C20—C24—H24A	109.5
C12—C11—H11B	108.8	C20—C24—H24B	109.5
C6—C11—H11B	108.8	H24A—C24—H24B	109.5
H11A—C11—H11B	107.7	C20—C24—H24C	109.5
C11—C12—C13	111.8 (2)	H24A—C24—H24C	109.5
C11—C12—H12A	109.2	H24B—C24—H24C	109.5
C13—C12—H12A	109.2	C26—C25—C19	116.9 (2)
C11—C12—H12B	109.2	C26—C25—H25A	108.1
C13—C12—H12B	109.2	C19—C25—H25A	108.1
H12A—C12—H12B	107.9	C26—C25—H25B	108.1
C12—C13—C14	113.05 (19)	C19—C25—H25B	108.1
C12—C13—C20	113.1 (2)	H25A—C25—H25B	107.3
C14—C13—C20	113.16 (18)	O6—C26—O5	124.2 (3)
C12—C13—H13	105.5	O6—C26—C25	124.1 (2)
C14—C13—H13	105.5	O5—C26—C25	111.6 (2)
C20—C13—H13	105.5	O5—C27—H27A	109.5
O4—C14—C15	102.50 (17)	O5—C27—H27B	109.5
O4—C14—C7	108.01 (18)	H27A—C27—H27B	109.5
C15—C14—C7	113.50 (19)	O5—C27—H27C	109.5
O4—C14—C13	110.14 (18)	H27A—C27—H27C	109.5
C15—C14—C13	109.10 (19)	H27B—C27—H27C	109.5
C7—C14—C13	113.05 (18)		
			
C2—O1—C1—C4	−0.5 (3)	O4—C14—C15—C16	45.6 (2)
C1—O1—C2—C3	0.7 (3)	C7—C14—C15—C16	161.85 (18)
O1—C2—C3—C4	−0.6 (3)	C13—C14—C15—C16	−71.1 (2)
O1—C1—C4—C3	0.1 (3)	C14—C15—C16—C21	77.3 (2)
O1—C1—C4—C5	179.1 (2)	C14—C15—C16—C17	−37.9 (2)
C2—C3—C4—C1	0.3 (3)	C14—O4—C17—C18	−111.5 (2)
C2—C3—C4—C5	−178.7 (2)	C14—O4—C17—C16	11.3 (2)
C9—O2—C5—C4	−159.5 (2)	C21—C16—C17—O4	−93.7 (2)
C9—O2—C5—C6	−32.0 (3)	C15—C16—C17—O4	17.8 (2)
C1—C4—C5—O2	−146.5 (2)	C21—C16—C17—C18	27.6 (3)
C3—C4—C5—O2	32.3 (3)	C15—C16—C17—C18	139.03 (19)
C1—C4—C5—C6	87.6 (3)	O4—C17—C18—C22	−59.7 (2)
C3—C4—C5—C6	−93.5 (3)	C16—C17—C18—C22	−178.62 (19)
O2—C5—C6—C10	−73.1 (2)	O4—C17—C18—C23	−177.11 (17)
C4—C5—C6—C10	47.7 (3)	C16—C17—C18—C23	63.9 (2)
O2—C5—C6—C11	167.36 (18)	O4—C17—C18—C19	58.9 (2)
C4—C5—C6—C11	−71.8 (2)	C16—C17—C18—C19	−60.1 (3)
O2—C5—C6—C7	48.6 (2)	C17—C18—C19—C25	153.7 (2)
C4—C5—C6—C7	169.41 (19)	C22—C18—C19—C25	−87.0 (2)
C10—C6—C7—C8	64.1 (2)	C23—C18—C19—C25	34.5 (3)
C11—C6—C7—C8	−176.87 (18)	C17—C18—C19—C20	25.4 (3)
C5—C6—C7—C8	−57.9 (2)	C22—C18—C19—C20	144.74 (19)
C10—C6—C7—C14	−170.21 (19)	C23—C18—C19—C20	−93.8 (2)
C11—C6—C7—C14	−51.2 (2)	C25—C19—C20—C21	−91.2 (2)
C5—C6—C7—C14	67.8 (2)	C18—C19—C20—C21	35.4 (2)
C14—C7—C8—C9	−77.0 (2)	C25—C19—C20—C24	27.7 (3)
C6—C7—C8—C9	50.6 (2)	C18—C19—C20—C24	154.2 (2)
C5—O2—C9—O3	−162.9 (2)	C25—C19—C20—C13	150.6 (2)
C5—O2—C9—C8	23.9 (3)	C18—C19—C20—C13	−82.8 (2)
C7—C8—C9—O3	153.6 (2)	C12—C13—C20—C21	−171.48 (18)
C7—C8—C9—O2	−33.5 (3)	C14—C13—C20—C21	−41.3 (3)
C10—C6—C11—C12	175.0 (2)	C12—C13—C20—C24	69.6 (3)
C5—C6—C11—C12	−64.2 (3)	C14—C13—C20—C24	−160.2 (2)
C7—C6—C11—C12	54.9 (3)	C12—C13—C20—C19	−56.2 (2)
C6—C11—C12—C13	−55.8 (3)	C14—C13—C20—C19	74.0 (3)
C11—C12—C13—C14	50.3 (2)	C15—C16—C21—O7	111.6 (3)
C11—C12—C13—C20	−179.43 (18)	C17—C16—C21—O7	−138.9 (2)
C17—O4—C14—C15	−36.1 (2)	C15—C16—C21—C20	−69.5 (2)
C17—O4—C14—C7	−156.17 (18)	C17—C16—C21—C20	39.9 (3)
C17—O4—C14—C13	79.9 (2)	C24—C20—C21—O7	−14.6 (3)
C8—C7—C14—O4	50.7 (2)	C19—C20—C21—O7	106.9 (3)
C6—C7—C14—O4	−73.6 (2)	C13—C20—C21—O7	−132.7 (2)
C8—C7—C14—C15	−62.3 (2)	C24—C20—C21—C16	166.6 (2)
C6—C7—C14—C15	173.51 (18)	C19—C20—C21—C16	−71.9 (2)
C8—C7—C14—C13	172.8 (2)	C13—C20—C21—C16	48.5 (2)
C6—C7—C14—C13	48.6 (3)	C18—C19—C25—C26	153.9 (2)
C12—C13—C14—O4	74.0 (2)	C20—C19—C25—C26	−77.3 (3)
C20—C13—C14—O4	−56.2 (3)	C27—O5—C26—O6	0.8 (4)
C12—C13—C14—C15	−174.24 (18)	C27—O5—C26—C25	−175.0 (2)
C20—C13—C14—C15	55.6 (3)	C19—C25—C26—O6	147.3 (3)
C12—C13—C14—C7	−46.9 (3)	C19—C25—C26—O5	−36.9 (3)
C20—C13—C14—C7	−177.13 (19)		
